# Network Pharmacology Analysis and Molecular Characterization of the Herbal Medicine Formulation Qi-Fu-Yin for the Inhibition of the Neuroinflammatory Biomarker iNOS in Microglial BV-2 Cells: Implication for the Treatment of Alzheimer's Disease

**DOI:** 10.1155/2020/5780703

**Published:** 2020-08-31

**Authors:** Fung Yin Ngo, Weiwei Wang, Qilei Chen, Jia Zhao, Hubiao Chen, Jin-Ming Gao, Jianhui Rong

**Affiliations:** ^1^School of Chinese Medicine, The University of Hong Kong, Hong Kong 999077, China; ^2^Shaanxi Key Laboratory of Natural Products & Chemical Biology, College of Chemistry & Pharmacy, Northwest A&F University, Yangling 712100, China; ^3^School of Chinese Medicine, Hong Kong Baptist University, Hong Kong 999077, China; ^4^Shenzhen Institute of Research and Innovation, The University of Hong Kong, Shenzhen 518057, China

## Abstract

Aberrant microglial activation drives neuroinflammation and neurodegeneration in Alzheimer's disease (AD). The present study is aimed at investigating whether the herbal formula Qi-Fu-Yin (QFY) could inhibit the inflammatory activation of cultured BV-2 microglia. A network pharmacology approach was employed to predict the active compounds of QFY, protein targets, and affected pathways. The representative pathways and molecular functions of the targets were analyzed by Gene Ontology (GO) and pathway enrichment. A total of 145 active compounds were selected from seven herbal ingredients of QFY. Targets (e.g., MAPT, APP, ACHE, iNOS, and COX-2) were predicted for the selected active compounds based on the relevance to AD and inflammation. As a validation, fractions were sequentially prepared by aqueous extraction, ethanolic precipitation, and HPLC separation, and assayed for downregulating two key proinflammatory biomarkers iNOS and COX-2 in lipopolysaccharide- (LPS-) challenged BV-2 cells by the Western blotting technique. Moreover, the compounds of QFY in 90% ethanol downregulated iNOS in BV-2 cells but showed no activity against COX-2 induction. Among the herbal ingredients of QFY, Angelicae Sinensis Radix and Ginseng Radix et Rhizoma contributed to the selective inhibition of iNOS induction. Furthermore, chemical analysis identified ginsenosides, especially Rg3, as antineuroinflammatory compounds. The herbal formula QFY may ameliorate neuroinflammation via downregulating iNOS in microglia.

## 1. Introduction

Alzheimer's disease (AD) is the major neurodegenerative cause of progressive dementia in the elderly [1]. The pathology of AD is hallmarked by the accumulation of extracellular *β*-amyloid (A*β*) and the formation of peptide plaques and intraneuronal tau lesions, resulting in impaired neurotransmission and neuronal death [2]. Among the existing pharmacological anti-AD interventions, acetylcholinesterase inhibitors are known to restore cholinergic neurotransmission, whereas *N*-methyl-D-aspartate (NMDA) receptor antagonists suppress the neuronal excitability towards NMDA [3]. However, some controversial results showed that these agents barely prevented the progression of AD and could cause adverse effects [4]. Nevertheless, neurotoxic peptide A*β* activates microglia and exacerbates neuroinflammation, leading to the onset and progression of AD [5, 6]. Indeed, the overexpression of proinflammatory enzymes such as inducible nitric oxide synthase (iNOS) and cyclooxygenase-2 (COX-2) jeopardizes the survival of neurons in brains [7]. Notably, excessive nitric oxide in the brain induced oxidative damage in neurons and led to the activation of apoptosis [8]. Therefore, effective inhibition of neuroinflammation represents a key strategy for the management of AD, but the in vivo efficacies of antineuroinflammatory and microglia-targeting agents remain uncertain [9].

Traditional herbal medicines may serve as alternative therapeutic strategies against various multifactorial and complex chronic diseases including AD [10]. The herbal formula Qi-Fu-Yin (QFY) was documented for managing dementia four hundred years ago [11]. The formula QFY is composed of seven herbal ingredients, namely, Atractylodis Macrocephalae Rhizoma (Baizhu, BZ), Angelicae Sinensis Radix (Danggui, DG), Glycyrrhizae Radix et Rhizoma (Gancao, GC), Ginseng Radix et Rhizoma (Renshen, RS), Rehmanniae Radix Preparata (Shudi, SD), Ziziphi Spinosae Semen (Suanzaoren, SZR), and Polygalae Radix (Yuanzhi, YZ). Previous studies showed that QFY improved learning and memory of AD rodents via increasing somatostatin in the hippocampus, reducing A*β* accumulation and proinflammatory biomarkers in mouse brains [12–14]. Comprehensive chemical profiling of QFY and a modified QFY formula with two additional herbs identified active compounds that were neuroprotective or exhibited a therapeutic effect towards AD [15, 16]. Several other studies also suggested the antineuroinflammatory action of GC, RS, and YZ via modulating microglial activity [17–19]. However, effort is needed to elucidate the molecular mechanisms by which QFY modulates microglial activation within the context of AD.

The aim of the present study was to characterize the antineuroinflammatory activity of QFY. We employed a network pharmacology approach to analyze the compound-target interactions of QFY and the relevant signaling pathways. We further validated the antineuroinflammatory property in microglial BV-2 cell culture and identified the principal active compounds through a bioactivity-guided fractionation procedure.

## 2. Materials and Methods

### 2.1. Chemicals and Reagents

The dried aqueous extracts of seven QFY ingredients were manufactured by Nong's Pharmaceutical Ltd., Hong Kong. Dulbecco's modified Eagle's medium (DMEM), fetal bovine serum (FBS), and penicillin/streptomycin solution were purchased from Invitrogen (Carlsbad, CA, USA). Protein assay dye reagent concentrate was purchased from Bio-Rad (Hercules, CA, USA). Lipopolysaccharide (LPS), RIPA assay buffer, protease inhibitor cocktail, and anti-rabbit horseradish peroxidase- (HRP-) conjugated IgG secondary antibody were purchased from Sigma-Aldrich (St. Louis, MO, USA). An antibody against iNOS was purchased from Abcam (Cambridge, UK). Antibodies against COX-2 and GAPDH were purchased from Cell Signaling Technology (Boston, MA, USA). Anti-mouse HRP-conjugated IgG was purchased from Santa Cruz Biotechnology (Santa Cruz, CA, USA). An enhanced chemiluminescence (ECL) detection reagent was purchased from GE Healthcare (Uppsala, Sweden). Ginsenoside Rg3 with the purity of >98% was purchased from Nanjing Spring and Autumn Biological Engineering Company (Nanjing, China).

### 2.2. Identification of the Active Compounds in the Formulation QFY

The chemical compounds were selected from the herbs of QFY in the databases of TCMSP (https://tcmspw.com/) based on the oral bioavailability (*OB*) ≥ 30%, drug‐likeness (*DL*) ≥ 0.18, and blood‐brain barrier (*BBB*) ≥ −0.3. On the other hand, the compounds in the herbal ingredient not found in TCMSP were selected from the database of TCMID (http://www.megabionet.org/tcmid/) when the compounds possessed the blood-brain barrier (BBB) permeability of ≥-1 through checking pkCSM (http://biosig.unimelb.edu.au/pkcsm/) and did not violate more than one of the criteria stated in Lipinski's rule of five. In addition, the active compounds were collected by text mining.

### 2.3. Prediction of Protein Targets

The active compounds in the formulation QFY were loaded to the Similarity Ensemble Approach (SEA) at http://sea.bkslab.org, the Search Tool for Interactions of Chemicals (STITCH) at http://stitch.embl.de, and SwissTargetPrediction at http://swisstargetprediction.ch for the prediction of the corresponding biological targets. The AD-related targets after checking Molecule Annotation System 3 (MAS 3.0) at http://bioinfo.capitalbio.com/mas3, Therapeutic Targets Database (TTD) at http://bidd.nus.edu.sg/group/cjttd/, and Comparative Toxicogenomics Database (CTD) at http://ctdbase.org/ were retained for further study.

### 2.4. Gene Ontology and Pathway Enrichment Analysis

The selected targets were analyzed for the enriched Gene Ontology (GO) terms and the Kyoto Encyclopedia of Genes and Genomes (KEGG) pathway using the online bioinformatics tool DAVID 6.8 at http://david.ncifcrf.gov. The annotations with adjusted *p* < 0.05 were considered significantly enriched.

### 2.5. Visualization of the Compound-Target Interaction and the Target-Pathway Relationship

The interaction networks were visualized using the open software package Cytoscape (http://www.cytoscape.org/). For the compound-target interactions, the active compounds in QFY and the corresponding protein targets were presented in a compound-target (C-T) network. For the target-pathway interactions, the targets and the related pathways were presented in a target-pathway (T-P) network.

### 2.6. Microglial BV-2 Cell Culture and Treatment

The murine microglial cell line BV-2 was purchased from the American Type Culture Collection (Manassas, VA, USA) and cultured in DMEM supplemented with 10% heat-inactivated FBS and 1% penicillin/streptomycin solution. The cells were incubated at an atmosphere with 5% CO_2_ at 37°C. For the indicated treatments, BV-2 cells were plated in 6-well plates at a density of 2 × 10^5^/mL overnight. BV-2 cells were treated with herbal extracts as indicated and costimulated with 0.1 *μ*g/mL LPS in DMEM supplemented with 3% FBS for 24 hours.

### 2.7. Fractionation of Herbal Extracts

QFY was prepared by mixing the dried aqueous extracts of seven herbal ingredients: BZ, 1.6 g; DG, 3 g; GC, 0.6 g; RS, 1.2 g; SD, 1.8 g; SZR, 1.2 g; and YZ, 1 g. Following extraction with distilled water at 80°C for 30 min, the mixture was cooled to room temperature and centrifuged at 13,000 rpm for 10 min. The supernatant was recovered and precipitated with ethanol at the final concentrations of 50%, 75%, and 90% overnight at 4°C. After centrifugation at 6,000 rpm for 30 min, the supernatant was collected to yield the corresponding ethanol solutions of QFY. The ethanolic materials were dried with a rotary evaporator, dissolved in DMSO, and sterilized by passing through a 0.22 *μ*m syringe filter for bioassays. For HPLC separation, the compounds were separated on an ACE C18 HPLC column (250 × 4.6 mm, 5 *μ*m) from Advanced Chromatography Technologies Ltd. (Aberdeen, Scotland, UK) under the control of the Waters Controller 600S HPLC system coupled with a photodiode array detector (Waters, Milford, MA, USA). The column temperature was maintained at 25°C. The mobile phases of (A) methanol and (B) 0.1% aqueous formic acid were pumped into the column at a flow rate of 1.0 mL/min to form the gradient as follows: 0-25 min, 5-50% A; 25-45 min, 50-70% A; 45-47 min, 70-95% A; and 47-53 min, 95% A. The fractions were collected, dried, and dissolved in DMSO for subsequent bioassays.

### 2.8. Western Blot Analysis

After drug treatment as indicated, BV-2 cells were washed twice with cold PBS and lysed with cold RIPA buffer to collect the cellular proteins. The cellular proteins (30 *μ*g) from each sample were resolved by 12% SDS-polyacrylamide gel electrophoresis and transferred to a polyvinylidene difluoride (PVDF) membrane. After overnight blocking with 5% non-fat milk, the membranes were probed with primary antibodies against iNOS, COX-2, and GAPDH overnight. The bound antibodies were detected with the HRP conjugate of the corresponding anti-rabbit or anti-mouse secondary antibodies for 2 hours. The activity of HRP was detected with ECL detection reagents. The blots were quantified by a densitometric method using the NIH ImageJ software.

### 2.9. Chemical Profiling by the LC-MS/MS System

The Agilent 6540 Ultra High Definition (UHD) Accurate-Mass Q-TOF LC/MS system from Agilent Technologies (Santa Clara, CA, USA) was used for mass spectrometry analysis. The compounds in 10 *μ*L were injected and separated on an ACE C18 HPLC column at a flow rate of 1.0 mL/min and column temperature of 40°C, by using the same gradient from the mobile phases of (A) 0.1% formic acid in water and (B) methanol as described for HPLC. The MS parameters were set as follows: electrospray ionization source (ESI) in a negative mode, nitrogen (N_2_) as drying gas, flow rate at 8 L/min, gas temperature at 300°C, nebulizer at 40 psi, sheath gas temperature at 350°C, flow rate of sheath gas at 8 L/min, capillary voltage at 4.0 kV, end plate offset at -500 V, fragmentor at 150 V, skimmer at 65 V, Oct RF Vpp at 600 V, scan range of 100-1700 m/z, and collision energy at 25 V for MS and 45 V for MS/MS, respectively. The data was analyzed on Agilent MassHunter Qualitative Analysis B.06.00 and Agilent MassHunter Quantitative Analysis B.06.00 from Agilent Technologies (Santa Clara, CA, USA).

### 2.10. Statistical Analysis

Data was presented as *mean* ± *SD* of three replicates. The difference between two groups was analyzed by one-way ANOVA and Tukey's multiple comparison using the GraphPad Prism 7 software (La Jolla, CA, USA). A *p* value of <0.05 was considered statistically significant.

## 3. Results

### 3.1. QFY Targets Key Proteins in Inflammation and AD Pathogenesis

**Figure 1:**
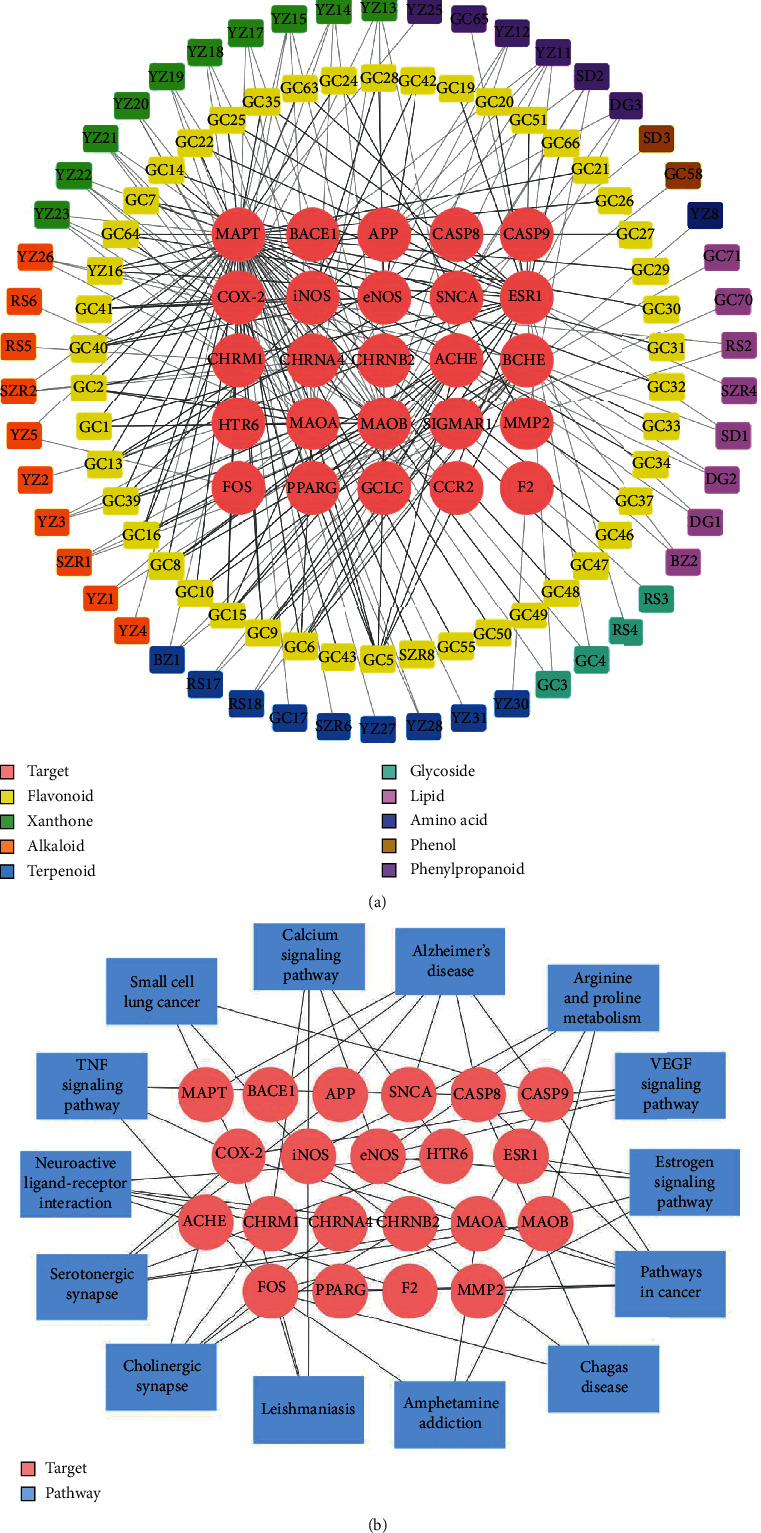
Network pharmacology analysis of QFY. (a) The compound-target (C-T) network. The grey lines indicate the interactions between the candidate active compounds (outer circles) from the individual herbal ingredients of QFY and the predicted protein targets at the center. (b) The target-pathway (T-P) network. The grey lines indicate the interactions between the protein targets at the center and the signaling pathways or diseases (outer circles).

To characterize the biological targets of QFY in a comprehensive manner, we employed a network pharmacology approach to dissect the complex multicompound and multitarget interaction. A total of 812 compounds in QFY were firstly retrieved from TCMSP and TCMID. Based on the pharmacokinetic or physiochemical properties, 126 active compounds were obtained while another 19 compounds were added by searching the PubMed database for the implications in the treatment of AD. The nomenclature of the active compounds was guided by PubChem, and the detailed information is listed in Supplementary Tables 1 and 2. The active compounds were further classified by the Medical Subject Headings (MeSH) classification system and text mining. The candidate compounds were chemically categorized into flavonoids, terpenoids, alkaloids, xanthones, phenylpropanoids, lipids, glycosides, carboxylic acids, phenolic compound, and amino acids. The protein targets were fished out using various predictive models (e.g., SEA, STITCH, and SwissTargetPrediction). As shown in Figure 1(a), 25 protein targets formed a total of 213 interactions with 96 active compounds. Specifically, MAPT as an important pathological target in AD showed the highest connectivity with 56 of the active compounds. Several active compounds in QFY showed interactions with other AD-relevant mediators (e.g., BACE1, APP, and SNCA), cholinergic neurotransmission (e.g., ACHE, CHRM1, CHRNA4, and CHRNB2), and inflammation (e.g., COX-2, eNOS, and iNOS). For the target-pathway network as shown in Figure 1(b), the protein targets were mapped to 14 different biological pathways or diseases in KEGG. Six protein targets (i.e., MAPT, BACE1, APP, SNCA, MAOA, and MAOB) were categorized in “Alzheimer's disease,” and five targets (i.e., ACHE, CHRM1, CHRNA4, CHRNB2, and FOS) were grouped to “cholinergic synapse,” while another three targets (i.e., CASP8, FOS, and COX-2) were grouped to “TNF signaling pathway”.

**Figure 2:**
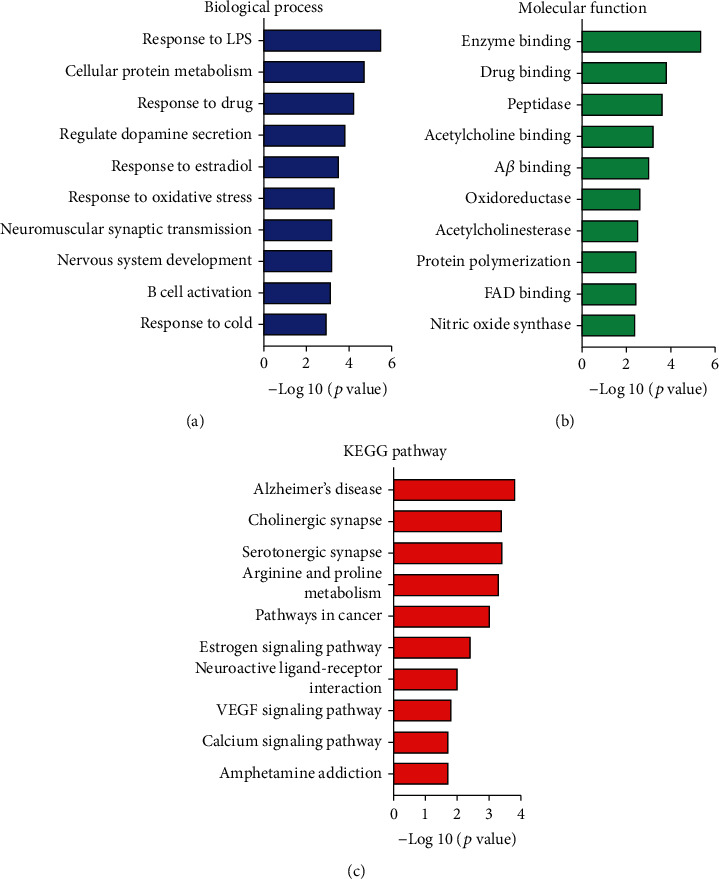
GO terms and KEGG pathway enrichment analysis of the protein targets. (a) The most significantly enriched GO terms were selected in terms of “biological process.” (b) The most significantly enriched GO terms were selected in terms of “molecular function.” (c) The most significantly enriched KEGG pathways were selected in terms of “KEGG pathways”.

To characterize the functional annotations of the protein targets, Gene Ontology (GO) and KEGG pathway enrichment analysis were carried out on the online bioinformatics tool DAVID. As shown in Figure 2, the inflammation- or AD-related GO terms appeared in the ten most significantly enriched GO terms from “biological process” and “molecular function,” in particular, acetylcholinesterase activity (GO: 0003990), amyloid-beta binding (GO: 0001540), and response to lipopolysaccharide (GO: 0032496). The three most significantly enriched KEGG pathways were Alzheimer's disease (KEGG: hsa05010), cholinergic neurotransmission pathway (KEGG: hsa04725), and serotonergic neurotransmission pathway (KEGG: hsa04726).

### 3.2. QFY Differentially Affected iNOS and COX-2 Expression against LPS Stimulation

**Figure 3:**
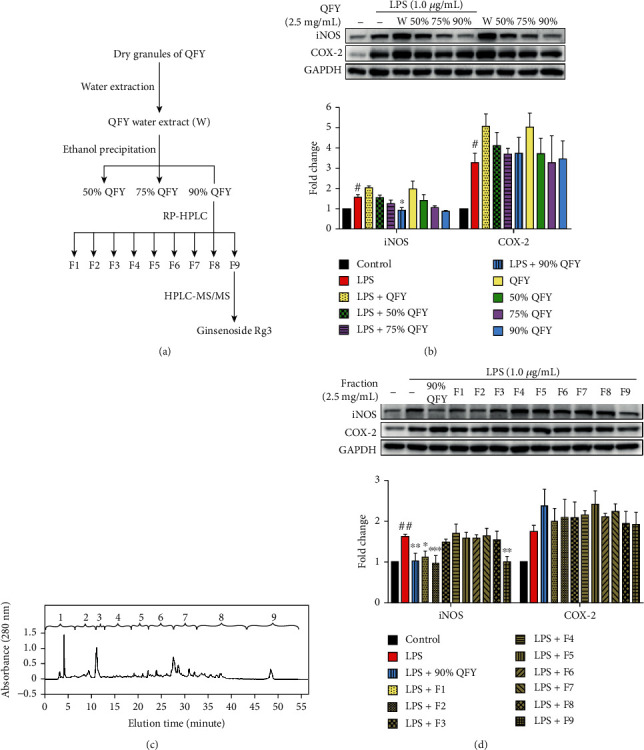
Bioactivity-guided fractionation of the herbal formulation QFY for potential active compounds. (a) Schematic illustration of the bioactivity-guided fractionation procedure. The fractions were prepared by sequential water extraction, ethanolic precipitation, and RP-HPLC separation on a C18 column. The fractions were sequentially analyzed by Western blot for the effects on LPS-induced expression of iNOS and COX-2 in BV-2 cells. (b) Bioassays of the QFY water extract and the fractions from ethanolic precipitation. BV-2 cells were treated with the indicated QFY extract with or without simultaneous stimulation with LPS for 24 h. The expression of iNOS and COX-2 was detected by Western blot using specific antibodies. Representative blots were shown. The blots (*n* = 3) were quantified by a densitometric method using the ImageJ software. The results were expressed as *mean* ± *SD*. ^#^*p* < 0.05 (LPS vs. untreated control); ^∗^*p* < 0.05 (treatment+LPS vs. LPS). (c) HPLC separation of the 90% QFY fraction. The compounds were separated into nine fractions by RP-HPLC on a C18 column. (d) Bioassays of the QFY-derived HPLC fractions. BV-2 cells were treated with the indicated concentration of different HPLC fractions or 90% QFY together with LPS stimulation. The cellular proteins were analyzed by Western blot, and the blots were quantified as previously described. ^##^*p* < 0.01 (LPS vs. untreated control); ^∗^*p* < 0.05, ^∗∗^*p* < 0.01, and ^∗∗∗^*p* < 0.001 (treatment+LPS vs. LPS).

To validate the antineuroinflammatory action of QFY and identify the corresponding active compounds, we developed a bioactivity-guided fractionation approach involving ethanolic precipitation, HPLC separation, and *in vitro* assays (Figure 3(a)). The aqueous extract of QFY was firstly precipitated in 50%, 75%, and 90% ethanol. The ethanol solutions and aqueous extract of QFY were assayed for the effects on iNOS and COX-2 in LPS-stimulated BV-2 cells. As shown in Figure 3(b), LPS-induced iNOS expression was decreased by these ethanol solutions of QFY. The 90% ethanol solution of QFY (90% QFY) selectively suppressed LPS-induced iNOS expression in a concentration-dependent manner. In contrast, none of the four QFY preparations effectively suppressed LPS-induced COX-2 expression. To identify the specific compounds, 90% QFY preparation was separated into nine fractions by reverse-phase HPLC on a C18 column (Figure 3(c)). Based on the results of Western blot analysis in Figure 3(d), fractions 1, 2, and 9 showed strong activity to downregulate LPS-induced iNOS expression. Interestingly, these active fractions showed comparable activity with the parent 90% QFY preparation, whereas the other fractions did not show much activity.

### 3.3. Ginsenosides in RS Were Identified as the Principal Active Compounds in QFY

**Figure 4:**
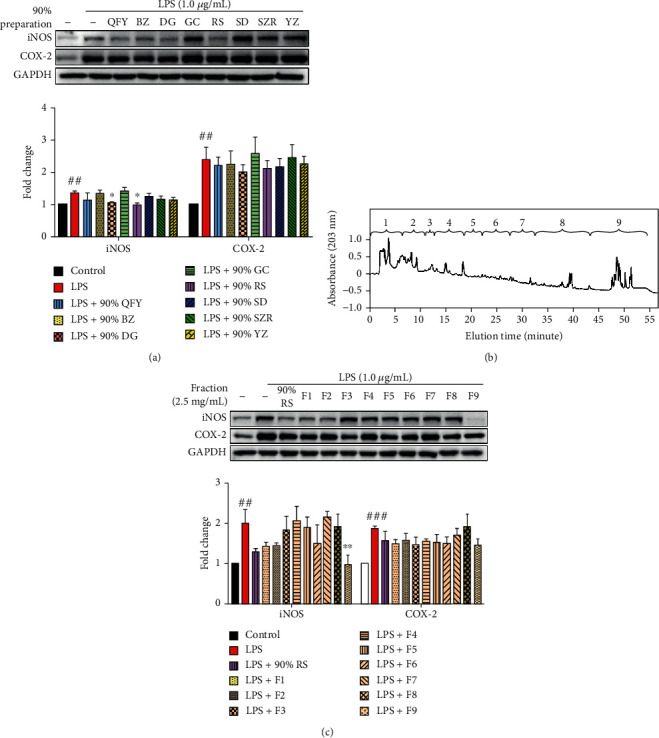
Identification of the active ingredients for the downregulation of iNOS expression. (a) Bioassays of the QFY extract and individual herbal extracts. QFY and individual herbal ingredients were extracted with water and precipitated with 90% ethanol. BV-2 cells were treated with 90% ethanol solutions of herbal extracts for 24 h with LPS stimulation. The expression of specific markers was detected by Western blot, and the blots were quantified as previously described. ^##^*p* < 0.01 (LPS vs. untreated control); ^∗^*p* < 0.05 (treatment+LPS vs. LPS). (b) HPLC separation of the 90% RS fraction. The 90% RS solution was separated into nine fractions by RP-HPLC on a C18 column using the same gradient and elution time as described for the 90% QFY fraction. (c) Bioassays of the 90% RS-derived HPLC fractions. BV-2 cells were treated with the indicated concentration of HPLC fractions or 90% RS for 24 h with LPS stimulation. The expression of specific markers was detected by Western blot, and the blots were quantified as previously described. ^##^*p* < 0.01, ^###^*p* < 0.001 (LPS vs. untreated control); ^∗∗^*p* < 0.01 (treatment+LPS vs. LPS).

To identify the active ingredients, seven herbal ingredients in QFY were processed into 90% ethanol solutions and assayed for downregulating iNOS in microglia following the procedure described for the whole QFY formulation. As shown in Figure 4(a), 90% ethanol extracts of DG (90% DG) and RS (90% RS) abolished LPS-induced iNOS expression but showed no effect against COX-2 induction. The 90% RS preparation appeared to be more potent than the 90% DG preparation, although less RS was used in the formula than DG. For this reason, the 90% RS solution was selected for further bioactivity-guided fractionation. The compounds in the 90% RS preparation were separated by HPLC under the same conditions as described for the 90% QFY preparation (Figure 4(b)). The resulting fractions were assayed for the activity towards iNOS induction. As shown in Figure 4(c), fraction 9 (90% RS-F9) effectively and selectively suppressed LPS-induced iNOS expression to a greater extent than the parent 90% RS solution.

**Figure 5:**
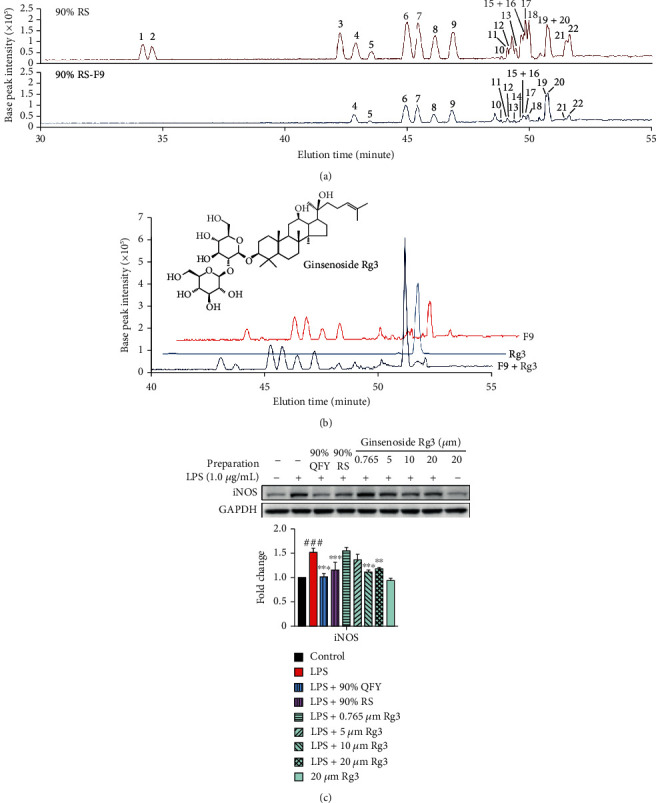
Chemical characterization of fraction 9 derived from 90% RS. (a) Chromatographic profiles of the parent 90% RS solution and the derived fraction 9. (b) Verification of ginsenoside Rg3 in the 90% RS-derived fraction 9. Fraction 9 and ginsenoside Rg3, alone or in combination, were analyzed by HPLC-MS/MS on a C18 column under the same conditions. (c) Bioassays of ginsenoside Rg3 for suppressing iNOS induction. BV-2 cells were treated with the indicated concentration of 90% QFY, 90% RS, and commercial ginsenoside Rg3 for 24 h. The expression of iNOS was detected by Western blot, and the blots were quantified as previously described. ^###^*p* < 0.001 (LPS vs. untreated control); ^∗∗^*p* < 0.01, ^∗∗∗^*p* < 0.001 (treatment+LPS vs. LPS).

To identify the specific ginsenosides, the 90% RS preparation and 90% RS-F9 were separated on a C18 HPLC column and detected with a MALDI-TOF mass spectrometer. As shown in Figure 5(a), the 90% RS-F9 fraction showed identical distribution of base peaks compared with the parent 90% RS solution within the elution time interval. As listed in Table 1, a total of 18 ginsenosides and one notoginsenoside were identified in the 90% RS-F9 fraction in a negative ion mode. In particular, peaks 19 and 20 were relatively prominent in the 90% RS-F9 fraction and detected as the stereoisomers of ginsenoside Rg3. 20(*R*)-Ginsenoside Rg3 and 20(*S*)-ginsenoside were mainly detected as formate ions at *m*/*z* 829.4965 and 829.4971, respectively, while the deprotonated ions were also detected at *m*/*z* 783.4906 and 783.7922, respectively. To compare the migration pattern with pure compounds, commercial ginsenoside Rg3 was spiked in the 90% RS-F9 fraction prior to LC-MS/MS analysis. As shown in Figure 5(b), ginsenoside Rg3 was verified by using the identical elution time and mass fragmentation pattern (data not shown). In addition, ginsenoside Rg3 was quantified from the acquired scan data using a target ion of 829.50. The calibration curve for ginsenoside Rg3 (*y* = 62240.86*x* − 24991.72, *R*^2^ = 0.9993) was optimized over the concentration range of 3.9–125 *μ*g/mL. The calculated amount of ginsenoside Rg3 in 90% RS and 90% RS-F9 was 2.11 ± 0.02 *mg*/*g* and 2.05 ± 0.08 *mg*/*g*, respectively.

To examine the potency of different preparations to downregulate iNOS expression, ginsenoside Rg3 and the parent extracts (i.e., 90% QFY and 90% RS) were normalized based on quantification results in the assay of the *in vitro* effects. As shown in Figure 5(c), 90% QFY and 90% RS suppressed iNOS induction to an extent similar to 10 *μ*M and 20 *μ*M of ginsenoside Rg3, respectively.

## 4. Discussion

Microglial activation triggers neuroinflammation and neurodegeneration in the onset of AD [20]. Single-drug therapies such as nonsteroidal anti-inflammatory drugs (NSAIDs), TNF-*α* inhibitor, advanced glycation end product receptor inhibitor, and PPAR*γ* receptor agonist show varying efficacies in the treatment of AD [21]. Recent effort is directed to the discovery of multitarget drugs by a systems biology approach [22]. Many herbal formulas are well documented for the clinical treatment of dementia in traditional Chinese medicine, thereby representing a rich source for drug discovery [23]. The present study investigated the classical herbal formula QFY for the active compounds and protein targets and hence the molecular mechanisms for the management of AD. We firstly employed a network pharmacology approach to predict the biological targets of QFY and the relevant signaling pathways. We further identified the active compounds from QFY for targeting the neuroinflammatory biomarker iNOS.

Network pharmacology encompasses systems biology, pharmacology, and computational algorithms to robustly study the complex drug-target relationships [24, 25]. Several herbal medicines including RS and YZ were previously studied for the molecular targets against AD by a network pharmacology approach [26]. In the present study, we employed a network pharmacology approach to study the action of the comprehensive anti-AD herbal formula QFY. Firstly, we shortlisted the active compounds with satisfactory bioavailability and BBB penetration based on the common pharmacokinetic or physiochemical parameters curated in databases. We secondly predicted 25 protein targets for these active compounds by target fishing. The C-T network in Figure 1(a) demonstrated that multiple compounds interacted with each protein, whereas the T-P network in Figure 1(b) also vividly depicted that the protein targets were involved in different pathways. Finally, the selected targets were subjected to GO and KEGG enrichment analysis. QFY may exert the pharmacological effect against AD by modulating A*β* aggregation, acetylcholinesterase activity, nitric oxide synthase activity, and cholinergic neurotransmission (Figure 2).

Neuroinflammation is well known to cause extensive damage to the central nervous system, leading to the disruption of synaptic function and the exacerbation of A*β* pathology [27]. Along this line, the present study focused on the antineuroinflammatory activity of QFY and selected two key proinflammatory biomarkers iNOS and COX-2 from the prediction of network pharmacology. It was previously demonstrated that both iNOS and COX-2 were overexpressed in the activated microglia and contributed to neurodegeneration [28]. To investigate the effect of QFY on the expression of iNOS and COX-2, we adopted a bioactivity-guided fractionation approach to separate QFY into different fractions by water extraction, ethanolic precipitation, and HPLC fractionation. We prepared the QFY formulation from the dried, concentrated granules of different herbal ingredients, which facilitated the extraction process and produced a similar chemical profile to regular decoction [29]. We assayed all fractions for the activity to suppress LPS-induced expression of iNOS and COX-2 in microglial BV-2 cells. Based on Western blot analysis, the aqueous QFY extract potentiated the stimulatory effect of LPS on the expression of iNOS and COX-2. The stimulatory effect on microglia could be attributed to the water-soluble active immunopolysaccharide in the herbal ingredients [30]. On the other hand, alcoholic precipitation removed charged small molecules and polysaccharides from the aqueous herbal extracts that could stimulate the cells [31, 32]. Indeed, the 90% QFY preparation selectively suppressed LPS-induced iNOS expression in microglial BV-2 cells.

A bioactivity-guided fractionation approach is widely used to separate complex chemical mixture into different fractions, facilitating the identification of active compounds for specific biological entities [33–35]. In the present study, we firstly prepared a water extract and 50%, 75%, and 90% QFY ethanol extracts. Based on the bioassay results, the 90% QFY preparation appeared to be the active fraction. Therefore, the 90% QFY preparation was subsequently separated into 9 fractions by HPLC (Figure 3(c)). Western blot analysis suggested that fractions 1, 2, and 9 effectively suppressed LPS-induced iNOS expression, although different chemicals might contribute to the activity (Figure 3(d)). For the identification of the active compounds, we screened seven herbal ingredients in the formulation QFY and identified that 90% DG and 90% RS preparations selectively suppressed iNOS induction in LPS-challenged BV-2 cells (Figure 4(a)). We selected the 90% RS preparation for further identification of the active compounds since RS-derived active compounds better met the requirements for an orally active drug in network pharmacology analysis. Thus, the 90% RS preparation was separated into 9 fractions by HPLC using the same gradient and fractionation as described for the 90% QFY preparation. Western blot analysis suggested that fraction 9 derived from 90% RS best suppressed LPS-induced iNOS expression (Figures 4(b) and 4(c)). The 90% RS and 90% QFY preparations showed similar efficacy of inhibiting iNOS expression. In fact, previous studies demonstrated that RS exhibited excellent antineuroinflammatory and anti-A*β* potency [17, 36]. Based on the LC-MS/MS profile, fraction 9 might contain several antineuroinflammatory ginsenosides such as Rh1, Rb2, Rd, Rg3, and Rg5 [37–40]. In particular, ginsenoside Rg3 could downregulate iNOS against LPS stimulation in macrophages and promote the phenotypic switch of macrophages towards a proresolving M2 subtype [41, 42]. Indeed, LC-MS/MS analysis confirmed the presence of ginsenoside Rg3 in 90% RS-derived fraction 9 since ginsenoside Rg3 spike-in comigrated with the endogenous ginsenoside Rg3 (Figure 5(b)). Furthermore, 10 *μ*M ginsenoside Rg3 appeared to achieve the same potency of 90% QFY and 90% RS for suppressing iNOS induction, although ginsenoside Rg3 failed to exhibit similar activity at the concentration in 90% RS as deduced by the UPLC quantification (Figure 5(c)). Presumably, other compounds may act on iNOS induction in synergy with ginsenoside Rg3. As for the differential effects of QFY fractions on LPS-induced expression of iNOS and COX-2, we previously found that the concomitant activation of the antioxidant Nrf2/HO-1 pathway was a potential mechanism to support the selective suppression of iNOS over COX-2 in macrophages [43]. As elevation of iNOS resulted in oxidative damage in neurons, activation of the Nrf2/HO-1 pathway could effectively inhibit inflammation and oxidative stress, which restored cellular function to the physiological state [44]. Previous studies reported that ginseng and ginsenoside Rg3 could activate the Nrf2/HO-1 pathway [45, 46]. We speculate that ginsenoside Rg3 selectively suppressed iNOS induction in microglia due to the concomitant activation of the Nrf2/HO-1 pathway.

## 5. Conclusions

The present study demonstrated that the active compounds of the herbal formulation QFY could target several protein targets and signaling pathways in the pathogenesis of AD. Ginsenoside Rg3 was identified for potential activity to suppress LPS-induced iNOS expression in BV-2 microglial cells. Ultimately, the present study confirmed the possibility to identify the active compounds for targeting the most important antineuroinflammatory biomarkers against microglial activation and towards the development of anti-AD therapeutics.

## Figures and Tables

**Table 1:** Chemicals identified from HPC chromatograms of 90% RS (from 30 to 55 min) and 90% RS-F9.

Peak	*t*_R_ (min)	Identification	Molecular formula	Major fragment				Other fragments	Possible source
				Theoretical *m*/*z*	Experimental *m*/*z*	Error (ppm)	Adduct ion		
1	34.149	Ginsenoside Re	C_48_H_82_O_18_	991.5483	991.5512	2.92	[M+HCOO]^−^	945.544 [M-H]^−799.4846^ [M-H-(Rha-H_2_O)]^−475.3788^ [M-H-(Rha-H_2_O)-2(Glc-H_2_O)]^−^	Roots
2	34.530	Ginsenoside Rg1	C_42_H_72_O_14_	845.4904	845.4911	0.83	[M+HCOO]^−^	799.5847 [M-H]^−637.4322^ [M-H-(Glc-H_2_O)]^−475.3792^ [M-H-2(Glc-H_2_O)]^−^	Roots
3	42.229	Gomisin P isomer	C_23_H_28_O_8_	431.1711	431.1720	2.09	[M-H]^−^	384.9360 [M-H-(CHO)-(H_2_O)]^−^	Metabolite
4	42.847	Ginsenoside Rf	C_42_H_72_O_14_	845.4904	845.4911	0.83	[M+HCOO]^−^	799.4680 [M-H]^−637.4319^ [M-H-(Glc-H_2_O)]^−475.3799^ [M-H-2(Glc-H_2_O)]^−^	Roots
5	43.490	Notoginsenoside R2	C_41_H_70_O_13_	815.4798	815.4802	0.49	[M+HCOO]^−^	769.4750 [M-H]^−637.4325^ [M-H-(Ara/Xyl-H_2_O)]^−475.3795^ [M-H-(Ara/Xyl-H_2_O)-(Glc-H_2_O)]^−^	Roots
6	44.903	20(*S*)-Ginsenoside Rg2	C_42_H_72_O_13_	829.4955	829.4969	1.69	[M+HCOO]^−^	783.4918 [M-H]^−637.4321^ [M-H-(Rha-H_2_O)]^−475.3800^ [M-H-(Rha-H_2_O)-(Glc-H_2_O)]^−^	Roots
7	45.431	20(*S*)-Ginsenoside Rh1	C_36_H_62_O_9_	683.4376	683.4383	1.02	[M+HCOO]^−^	637.4325 [M + cl]^−475.3789^ [M-H-(Glc-H_2_O)]^−^	Roots
8	46.106	20(*R*)-Ginsenoside Rg2	C_42_H_72_O_13_	829.4955	829.4969	1.69	[M+HCOO]^−^	783.4916 [M-H]^−637.4325^ [M-H-(Rha-H_2_O)]^−475.3798^ [M-H-(Rha-H_2_O)-(Glc-H_2_O)]^−^	Roots
9	46.868	20(*R*)-Ginsenoside Rh1	C_36_H_62_O_9_	683.4376	683.4383	1.02	[M+HCOO]^−^	637.4322 [M + cl]^−475.3796^ [M-H-(Glc-H_2_O)]^−^	Roots
10	48.833	Ginsenoside Ro	C_48_H_76_O_19_	955.4908	955.4914	0.63	[M-H]^−^	793.4452 [M-H-(Glc-H_2_O)]^−^	Roots
11	49.094	Ginsenoside Rb1	C_54_H_92_O_23_	1153.6011	1153.6010	0.26	[M+HCOO]^−^	1107.5979 [M-H]^−945.5461^ [M-H-(Glc-H_2_O)]^−783.4906^ [M-H-2(Glc-H_2_O)]^−^	Roots
12	49.180	Ginsenoside Rc	C_53_H_90_O_22_	1123.5906	1123.5920	0.80	[M+HCOO]^−^	1077.5878 [M-H]^−945.5437^ [M-H-(Ara(f)-H_2_O)]^−783.4902^ [M-H-(Glc-H_2_O)-(Ara(f)-H_2_O)]^−^	Roots
13	49.419	Ginsenoside Rb2	C_53_H_90_O_22_	1123.5906	1123.5920	0.80	[M+HCOO]^−^	1077.5878 [M-H]^−945.5437^ [M-H-(Ara(p)-H_2_O)]^−783.4902^ [M-H-(Glc-H_2_O)-(Ara(p)-H_2_O)]^−^	Roots
14	49.654	Ginsenoside Rg6	C_42_H_70_O_12_	811.4849	811.4868	2.34	[M+HCOO]^−^	765.4806 [M-H]^−619.4227^ [M-H-(Rha-H_2_O)]^−457.3683^ [M-H-(Glc-H_2_O)-(Rha-H_2_O)]^−^	Steamed roots
15	49.742	Ginsenoside Rd	C_48_H_82_O_18_	991.5483	991.5506	2.32	[M+HCOO]^−^	945.5441	Steamed roots
16	49.771	Ginsenoside Rk3	C_36_H_60_O_8_	665.4270	665.4280	1.50	[M+HCOO]^−^	619.4262 [M-H]^−^	Steamed roots
17	49.801	Ginsenoside F4	C_42_H_70_O_12_	811.4849	811.4855	0.74	[M+HCOO]^−^	765.4807 [M-H]^−619.4224^ [M-H-(Rha-H_2_O)]^−457.3696^ [M-H-(Glc-H_2_O)-(Rha-H_2_O)]^−^	Leaves
18	49.947	Ginsenoside Rh4	C_36_H_60_O_8_	665.4270	665.4280	1.50	[M+HCOO]^−^	619.4205 [M-H]^−^	Steamed roots
19	50.681	20(*S*)-Ginsenoside Rg3	C_42_H_72_O_13_	829.4955	829.4971	1.93	[M+HCOO]^−^	783.7922 [M-H]^−621.4373^ [M-H-(Glc-H_2_O)]^−459.3856^ [M-H-(Glc-H_2_O)]^−^	Steamed roots
20	50.768	20(*R*)-Ginsenoside Rg3	C_42_H_72_O_13_	829.4955	829.4965	1.21	[M+HCOO]^−^	783.4906 [M-H]^−621.4386^ [M-H-(Glc-H_2_O)]^−459.3847^ [M-H-(Glc-H_2_O)]^−^	Steamed roots
21	51.472	Ginsenoside Rk1	C_42_H_70_O_12_	811.4849	811.4848	-0.12	[M+HCOO]^−^	765.4803 [M-H]^−603.4266^ [M-H-(Glc-H_2_O)]^−^	Steamed roots
22	51.619	Ginsenoside Rg5	C_42_H_70_O_12_	811.4849	811.4868	2.34	[M+HCOO]^−^	765.4806 [M-H]^−603.4276^ [M-H-(Glc-H_2_O)]^−^	Steamed roots

Rha: *α*-L-rhamnose; Glc: *β*-D-glucose; Ara(p): *α*-L-arabinose (pyranose); Ara(f): *α*-L-arabinose (furanose); Xyl: *β*-D-xylose.

## Data Availability

The data supporting the findings of this study are available in the article and its supplementary materials.

## Supplementary Materials

Supplementary Materials Table S1: Candidate compounds obtained from the TCMSP database and their pharmacokinetic parameters. Table S2: Candidate compounds obtained from the TCMID database and their Lipinski's rule of five parameters.
