# Supplementary material for: Network Pharmacology Analysis and Molecular Characterization of the Herbal Medicine Formulation Qi-Fu-Yin for the Inhibition of the Neuroinflammatory Biomarker iNOS in Microglial BV-2 Cells: Implication for the Treatment of Alzheimer's Disease
Source: Oxid Med Cell Longev. 2020 Aug 31;2020:5780703. doi: 10.1155/2020/5780703 (PMC7481926; doi:10.1155/2020/5780703)
Supplement: Supplementary Materials — Table S1: Candidate compounds obtained from the TCMSP database and their pharmacokinetic parameters. Table S2: Candidate compounds obtained from the TCMID database and their Lipinski's rule of five parameters. [file 5780703.f1.docx]

Table S1. Candidate compounds obtained from TCMSP database and their pharmacokinetic parameters. OB, oral bioavailability; DL, drug-likeness; BBB, blood brain barrier.

| No. | Name | Structure | OB (%) | DL | BBB |
| --- | --- | --- | --- | --- | --- |
| BZ1 | α-Amyrin |  | 39.51 | 0.22 | 1.28 |
| BZ2 | (24S)-24-Propylcholesta-5-ene-3-beta-ol |  | 36.23 | 0.31 | 1.09 |
| BZ3 | 3β-Acetoxyatractylone |  | 54.07 | 0.3 | 1.08 |
| BZ4 | 8β-Ethoxyatractylenolide Ⅲ |  | 35.95 | 0.76 | 1.12 |
| BZ5 | Atractylenolide III |  | 31.66 | 0.17 | 0.64 |
| DG1 | β-Sitosterol |  | 36.91 | 0.75 | 0.99 |
| DG2 | Stigmasterol |  | 43.83 | 0.76 | 1.00 |
| DG3 | Ferulic acid |  | 39.56 | 0.06 | -0.03 |
| DG4 | (Z)-Butylidenephthalide |  | 42.22 | 0.07 | 1.25 |
| DG5 | (Z)-Ligustilide |  | 23.5 | 0.07 | 1.20 |
| DG6 | (3S)-Butylphthalide |  | - | - | - |
| GC1 | S-Liquiritigenin |  | 32.76 | 0.18 | -0.29 |
| GC2 | (2R)-7-Hydroxy-2-(4-hydroxyphenyl)-chroman-4-one |  | 71.12 | 0.18 | -0.25 |
| GC3 | Liquiritin |  | 65.69 | 0.74 | -1.93 |
| GC4 | Liquiritin |  | 65.69 | 0.74 | -1.93 |
| GC5 | Glabranin |  | 52.9 | 0.31 | 0.31 |
| GC6 | Isobavachin |  | 36.57 | 0.32 | -0.04 |
| GC7 | Glepidotin A |  | 44.72 | 0.35 | 0.06 |
| GC8 | Glepidotin B |  | 64.46 | 0.34 | -0.09 |
| GC9 | Euchrenone |  | 30.29 | 0.57 | 0.39 |
| GC10 | 6-Prenyleriodictyol |  | 39.22 | 0.41 | -0.29 |
| GC11 | Shinflavanone |  | 31.79 | 0.72 | 0.25 |
| GC12 | Xambioona |  | 54.85 | 0.87 | 0.52 |
| GC13 | Formononetin |  | 69.67 | 0.21 | 0.02 |
| GC14 | Isoformononetin |  | 38.37 | 0.21 | 0.25 |
| GC15 | 7-Methoxy-2-methylisoflavone |  | 42.56 | 0.2 | 0.56 |
| GC16 | 7-Acetoxy-2-methylisoflavone |  | 38.92 | 0.26 | 0.16 |
| GC17 | Odoratin |  | 49.95 | 0.3 | -0.24 |
| GC18 | Licoricone |  | 63.58 | 0.47 | -0.14 |
| GC19 | Eurycarpin A |  | 43.28 | 0.37 | -0.06 |
| GC20 | Licoisoflavone A |  | 41.61 | 0.42 | -0.27 |
| GC21 | Gancaonin M |  | 30.49 | 0.41 | 0.21 |
| GC22 | Lupiwighteone |  | 51.64 | 0.37 | -0.23 |
| GC23 | Gancaonin L |  | 66.37 | 0.41 | -0.13 |
| GC24 | Gancaonin O |  | 44.15 | 0.41 | -0.28 |
| GC25 | Gancaonin A |  | 51.08 | 0.4 | 0.13 |
| GC26 | Gancaonin B |  | 48.79 | 0.45 | -0.10 |
| GC27 | Glyasperin B |  | 65.22 | 0.44 | -0.09 |
| GC28 | Dehydroglyasperins C |  | 53.82 | 0.37 | -0.12 |
| GC29 | Gancaonin G |  | 60.44 | 0.39 | 0.30 |
| GC30 | Gancaonin H |  | 50.1 | 0.78 | -0.14 |
| GC31 | Glabrone |  | 52.51 | 0.5 | -0.11 |
| GC32 | Licoisoflavone B |  | 38.93 | 0.55 | -0.18 |
| GC33 | Licoisoflavanone |  | 52.47 | 0.54 | -0.22 |
| GC34 | Glyasperin M |  | 72.67 | 0.59 | -0.04 |
| GC35 | Kumatakenin |  | 50.83 | 0.29 | -0.22 |
| GC36 | Glyasperin F |  | 75.84 | 0.54 | -0.15 |
| GC37 | Glyzaglabrin |  | 61.07 | 0.35 | -0.20 |
| GC38 | Licoagroisoflavone |  | 57.28 | 0.49 | 0.09 |
| GC39 | Glypallichalcone |  | 61.6 | 0.19 | 0.23 |
| GC40 | Licochalcone A |  | 40.79 | 0.29 | -0.21 |
| GC41 | Licochalcone G |  | 49.25 | 0.32 | -0.04 |
| GC42 | Kanzonol B |  | 39.62 | 0.35 | -0.12 |
| GC43 | Vestitol |  | 74.66 | 0.21 | 0.30 |
| GC44 | Glyasperin C |  | 45.56 | 0.4 | 0.07 |
| GC45 | Glabrene |  | 46.27 | 0.44 | 0.04 |
| GC46 | Phaseolinisoflavan |  | 32.01 | 0.45 | 0.46 |
| GC47 | Glabridin |  | 53.25 | 0.47 | 0.36 |
| GC48 | 4'-Methoxyglabridin |  | 36.21 | 0.52 | 0.61 |
| GC49 | 3'-Hydroxy-4'-O-methylglabridin |  | 43.71 | 0.57 | 0.73 |
| GC50 | 3'-Methoxyglabridin |  | 46.16 | 0.57 | 0.47 |
| GC51 | Medicarpin |  | 49.22 | 0.34 | 0.53 |
| GC52 | 1-Methoxyphaseollidin |  | 69.98 | 0.64 | 0.48 |
| GC53 | Licoagrocarpin |  | 58.81 | 0.58 | 0.61 |
| GC54 | Inermine |  | 75.18 | 0.54 | 0.40 |
| GC55 | Shinpterocarpin |  | 80.3 | 0.73 | 0.68 |
| GC56 | Kanzonol F |  | 32.47 | 0.89 | 0.56 |
| GC57 | Kanzonol U |  | 58.44 | 0.38 | 0.34 |
| GC58 | Licocoumarone |  | 33.21 | 0.36 | 0.06 |
| GC59 | Inflacoumarin A |  | 39.71 | 0.33 | -0.24 |
| GC60 | Glycyrin |  | 52.61 | 0.47 | -0.13 |
| GC61 | Licoarylcoumarin |  | 59.62 | 0.43 | -0.23 |
| GC62 | Kanzonol W |  | 50.48 | 0.52 | -0.04 |
| GC63 | Isotrifoliol |  | 31.94 | 0.42 | -0.25 |
| GC64 | Hedysarimcoumestan B |  | 48.14 | 0.43 | -0.19 |
| GC65 | Glycyrol |  | 90.78 | 0.67 | -0.20 |
| GC66 | Phaseol |  | 78.77 | 0.58 | -0.04 |
| GC67 | Isoglycyrol |  | 44.7 | 0.84 | 0.05 |
| GC68 | Glycyrrhizin |  | 19.62 | 0.11 | -2.86 |
| GC69 | Glycyrrhetinic acid |  | 22.05 | 0.74 | -0.53 |
| GC70 | Icos-5-enoic acid |  | 30.7 | 0.2 | 1.09 |
| GC71 | Gadelaidic acid |  | 30.7 | 0.2 | 0.94 |
| RS1 | Diisooctyl phthalate |  | 43.59 | 0.39 | 0.26 |
| RS2 | Arachidonate |  | 45.57 | 0.2 | 0.58 |
| RS3 | Panaxadiol |  | 33.09 | 0.79 | 0.23 |
| RS4 | Ginsenoside-Rh4_qt |  | 31.11 | 0.78 | -0.18 |
| RS5 | Aponorhyoscine |  | 66.65 | 0.22 | 0.40 |
| RS6 | Celabenzine |  | 101.88 | 0.49 | 0.05 |
| RS7 | Deoxyharringtonine |  | 39.27 | 0.81 | -0.25 |
| RS8 | Girinimbine |  | 61.22 | 0.31 | 1.22 |
| RS9 | Gomisin B |  | 31.99 | 0.83 | 0.18 |
| RS10 | Malkangunin |  | 57.71 | 0.63 | -0.17 |
| RS11 | Suchilactone |  | 57.52 | 0.56 | 0.28 |
| RS12 | Frutinone A |  | 65.9 | 0.34 | 0.46 |
| RS13 | Inermine |  | 65.83 | 0.54 | 0.36 |
| RS14 | Ginsenoside Rb1 |  | 6.29 | 0.04 | -4.95 |
| RS15 | Ginsenoside Rd |  | - | - | - |
| RS16 | Ginsenoside Rg1 |  | - | - | - |
| RS17 | Protopanaxadiol |  | - | - | - |
| RS18 | Protopanaxatriol |  | - | - | - |
| SD1 | Sitosterol |  | 36.91 | 0.75 | 0.87 |
| SD2 | Caffeic acid |  | 25.76 | 0.05 | -0.26 |
| SD3 | Hydroxytyrosol |  | 57.57 | 0.03 | -0.17 |
| SD4 | Stigmasterol |  | 43.83 | 0.76 | 1.00 |
| SZR1 | Coclaurine |  | 42.35 | 0.7 | 0.06 |
| SZR2 | Zizyphusine |  | 41.53 | 0.99 | 0.60 |
| SZR3 | Daucosterol |  | 36.91 | 1.42 | 1.15 |
| SZR4 | Sitosterol |  | 36.91 | 1.43 | 1.16 |
| SZR5 | Jujuboside A_qt |  | 34.96 | 0.59 | 0.11 |
| SZR6 | Betulinic acid |  | 55.38 | 0.73 | 0.22 |
| SZR7 | Sanjoinenine |  | 67.28 | 0.43 | -0.24 |
| SZR8 | Spinosin |  | 6.31 | 0.72 | -3.29 |

**Table S2.** Candidate compounds obtained from TCMID database and their Lipinski’s rule of five parameters. MW, molecular weight; logP, partition coefficient; HBA, number of hydrogen bond acceptors; HBD, number of hydrogen bond donors; BBB, blood brain barrier.

| No. | Molecule name | Structure | MW | logP | HBA | HBD | BBB |
| --- | --- | --- | --- | --- | --- | --- | --- |
| YZ1 | Norharman |  | 170.19 | 3.2 | 1 | 1 | 0.20 |
| YZ2 | Harman |  | 182.23 | 3.6 | 1 | 1 | 0.21 |
| YZ3 | Harmine |  | 211.25 | 3.6 | 2 | 1 | 0.42 |
| YZ4 | Methyl β-carboline-3-carboxylate |  | 226.24 | 2.5 | 3 | 2 | 0.79 |
| YZ5 | 1-Methoxycarbonyl-beta-carboline |  | 240.26 | 2.9 | 3 | 1 | 0.78 |
| YZ6 | β-Carboline-1-propanoic acid |  | 240.26 | 2.1 | 3 | 2 | 0.29 |
| YZ7 | Perlolyrine |  | 264.28 | 2.2 | 3 | 2 | 0.41 |
| YZ8 | S-(2-carboxyethyl)-L-cysteine |  | 193.22 | -3.2 | 6 | 3 | -0.88 |
| YZ9 | 2-((Carboxymethyl) amino)-4-chlorobenzoic acid |  | 229.62 | 3.6 | 5 | 3 | -0.89 |
| YZ10 | N-Acetyl-D-glucosamine |  | 234.21 | -3.4 | 7 | 6 | -0.71 |
| YZ11 | 3,4-Dimethoxycinnamic acid |  | 208.21 | 1.8 | 4 | 1 | 0.50 |
| YZ12 | 3,4,5-Trimethoxycinnamic acid |  | 238.24 | 1.4 | 5 | 1 | -0.17 |
| YZ13 | 1,7-Dimethoxyxanthone |  | 256.26 | 3.3 | 4 | 0 | -0.004 |
| YZ14 | 1,7-Dihydroxy-3-methoxyxanthone |  | 258.23 | 2.8 | 5 | 2 | -0.28 |
| YZ15 | 1-Hydroxy-3,7-dimethoxyxanthone |  | 272.26 | 3.1 | 5 | 1 | -0.33 |
| YZ16 | Geraldone |  | 284.27 | 3.2 | 5 | 2 | -0.31 |
| YZ17 | 1,6-Dihydroxy-3,7-dimethoxyxanthone |  | 288.26 | 2.7 | 2 | 6 | -0.56 |
| YZ18 | 1,3-Dihydroxy-4,5-dimethoxyxanthone |  | 288.26 | 2.7 | 6 | 2 | -0.54 |
| YZ19 | 1-Hydroxy-3,6,7-trimethoxyxanthone |  | 302.28 | 3.1 | 6 | 1 | -0.66 |
| YZ20 | Onjixanthone I |  | 302.28 | 2.5 | 6 | 1 | -0.59 |
| YZ21 | 1,6-Dihydroxy-3,5,7-trimethoxyxanthone |  | 318.28 | 2.7 | 7 | 2 | -0.81 |
| YZ22 | 6-Hydroxy-1 ,2,3,7-tetramethoxyxanthone |  | 332.31 | 2.5 | 7 | 1 | -0.91 |
| YZ23 | 1,2,3,6,7-Pentamethoxyxanthone |  | 346.34 | 2.8 | 7 | 0 | -0.97 |
| YZ24 | Polygalitol |  | 164.16 | -2.1 | 5 | 4 | -0.59 |
| YZ25 | 5,6,7-Trimethoxycoumarin |  | 236.22 | 1.8 | 5 | 0 | -0.08 |
| YZ26 | Norhyoscyamine |  | 275.35 | 4 | 2 | 1.4 | -0.16 |
| YZ27 | Tenulin |  | 306.36 | 0.3 | 5 | 1 | -0.15 |
| YZ28 | 1-Peroxyferolide |  | 338.36 | 0.9 | 7 | 1 | -0.71 |
| YZ29 | CHEMBL1077894 |  | 296.32 | 2.8 | 4 | 1 | 0.07 |
| YZ30 | Tenuifolin |  | 680.83 | 2.2 | 12 | 8 | -1.34 |
| YZ31 | Tenuigenin |  | 537.13 | 5.1 | 6 | 4 | -0.78 |
| YZ32 | Tenuifoliside B |  | 668.60 | -0.9 | 17 | 8 | -2.05 |
| YZ33 | (5-Formylfuran-2-yl) methyl 4-hydroxy-2-methylenebutanoate |  | 224.21 | 1.1 | 5 | 1 | -0.47 |
